# *Miscanthus*-Derived Energy Storage System Material Production

**DOI:** 10.1021/acsomega.3c00024

**Published:** 2023-02-22

**Authors:** Fikret Muge Alptekin, Nurhan Turgut Dunford, Melih Soner Celiktas

**Affiliations:** † Ege University, Solar Energy Institute, Izmir 35040, Turkey; ‡ Department of Biosystems and Agricultural Engineering, Oklahoma State University, Stillwater, Oklahoma 74078, United States

## Abstract

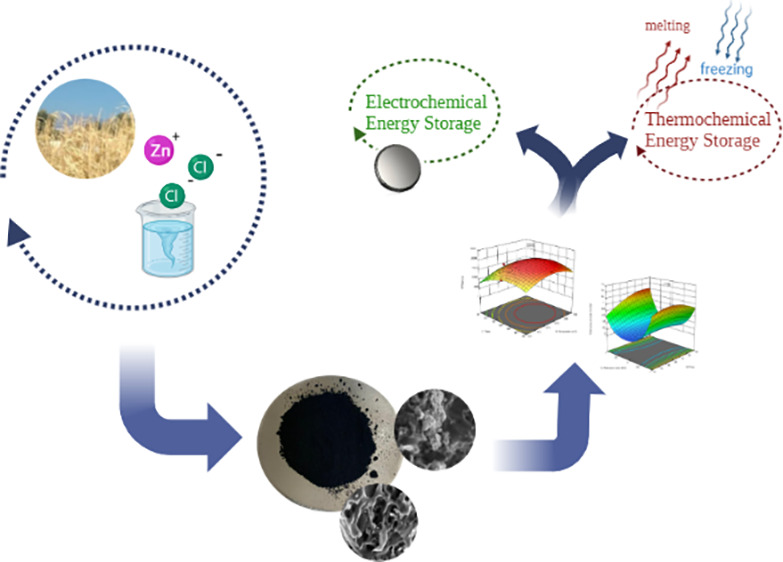

Carbon derived from various biomass sources has been evaluated as support material for thermal energy storage systems. However, process optimization of *Miscanthus*-derived carbon to be used for encapsulating phase change materials has not been reported to date. In this study, process optimization to evaluate the effects of selected operation parameters of pyrolysis time, temperature, and biomass:catalyst mass ratio on the surface area and pore volume of produced carbon is conducted using response surface methodology. In the process, ZnCl_2_ is used as a catalyst to promote high pore volume and area formation. Two sets of optimum conditions with different pyrolysis operation parameters in order to produce carbons with the highest pore area and volume are determined as 614 °C, 53 min, and 1:2 biomass to catalyst ratio and 722 °C, 77 min, and 1:4 biomass to catalyst ratio with 1415.4 m^2^/g and 0.748 cm^3^/g and 1499.8 m^2^/g and 1.443 cm^3^/g total pore volume, respectively. Carbon material produced at 614 °C exhibits mostly micro- and mesosized pores, while carbon obtained at 722 °C comprises mostly of meso- and macroporous structures. Findings of this study demonstrate the significance of process optimization for designing porous carbon material to be used in thermal and electrochemical energy storage systems.

## 1 Introduction

Detrimental effects of greenhouse gas emissions and increasing price of fossil fuels have accelerated the search for alternative energy resources, cleaner feedstock conversion technologies and promoted efficient use of energy.^1−3^ Although renewable energy resources are clean and abundant, they have natural disadvantages including intermittency and randomness that cause incompatibility between supply and demand.^4^

Thermal energy storage (TES) and electrochemical energy storage (EES) are two types of energy storage systems that can be suitable for meeting the instant energy demand from renewable sources as needed. For instance, TES is used to store heat from solar radiation, waste heat, and gas boilers, while EES stores electricity through supercapacitors and batteries.^5^ TES has an important role to play in renewable energy storage systems.^6^ Among the other TES technologies, latent heat storage based on phase change materials (PCM) are considered to be the most promising technology due to their high energy storage density and cost effectiveness.^7−10^ Although liquid–solid PCM based TES has many advantages, leakage of the PCM out of the supporting material during its phase transition is a problem that requires further research and development work for improvement.^11,12^ Biomass-based highly porous carbon materials can be used to encapsulate PCM to address the leakage problem.

High power and energy density, long time stability during charging/discharging cycles, and safety are the key elements required in high performance electrochemical energy storage systems including lithium and sodium-ion batteries and supercapacitors.^13^ Carbon-based materials are considered as suitable electrode materials for supercapacitors and lithium-ion batteries due to their outstanding properties including, high electrical conductivity, chemical stability, and high surface area and pore volume.^14,15^

Biomass derived carbon materials have been utilized in a variety of applications such as soil and wastewater remediation, recovery of plant growth nutrients nitrogen and phosphorous from agricultural runoffs, and adsorption of heavy metals and other pollutants from wastewater streams,^16^ as electrodes in supercapacitors, and PCM support materials.^17^ However, further research and development work is necessary to develop carbon materials with high specific surface area, pore volume, and thermal conductivity to enhance their functionality in TES systems. Physical, chemical, and physicochemical activation methods have been used to increase the porosity and specific area of the biochar to be used in TES applications.^18^ The activation method, impregnation time, process temperature, residence time, and chemical composition of the biomass have significant effects on the final product characteristics.^19^ H_3_PO_4_, ZnCl_2_,^20^ KOH,^21^ NaOH, K_2_CO_3_,^22^ and Na_2_CO_3_ are chemicals that are widely used in the chemical activation processes. Among the latter chemicals, ZnCl_2_ is shown to be very effective in decomposing cellulosic material and produce carbon materials with high surface area and desirable pore structure.^23^ In Table S1, various biomass precursors, chemical agents used for activation, and process conditions for producing carbon material are summarized. *Miscanthus* has been examined as one of the promising biomass types for biofuel and biochar production in some countries, especially in the EU and USA.^24^

Biomass-based activated carbon having high specific surface area (SSA) and pore volume, with a physical structure consisting of micro-, meso-, and macrosized pores and high electrical conductivity, is considered to be a viable material for energy storage systems.^25,26^ However, the scientific literature lacks data on the optimization of the process parameters to attain the highest specific surface area and pore volume in carbon materials produced from different biomass types. Considering the very complex chemical and physical characteristics of biomass from different sources, process optimization is the key to evaluate viability of any biomass as feedstock to develop effective PCM support material and carbon material to be used in TES and EES systems. Response surface methodology (RSM) is a statistical method that is frequently used to determine the optimum conditions for a set of process variables and their interactions using a proper experimental design.^27−29^

In this study, optimization of process variables for producing *Miscanthus*-derived carbon materials through ZnCl_2_ activation was aimed in order to investigate their use as energy storage material. RSM was used to determine the optimum pyrolysis conditions to produce *Miscanthus*-based carbon material with high specific surface area and pore volume. The effect of ZnCl_2_ as a chemical activator in pore formation during was also evaluated to produce a material that can be suitable for both TES and EES systems. The range of the process variables examined in this was as follows: temperature (450–750 °C), residence time (30–90 min), and ZnCl_2_ impregnation ratio (biomass:ZnCl_2_ weight ratio) (1,0, 1:2, 1:4). The optimum process conditions determined via RSM were also confirmed by experiments. Physicochemical properties of the activated carbon generated were characterized using standard imaging and analytical techniques.

## 2 Material and Methods

### 2.1 Materials

The dry *Miscanthus* sample used in this study was provided by the University of Illinois (IL, USA). ZnCl_2_ (98.5% purity) was purchased from Acros Organics (Thermo Fisher Scientific, Waltham, MA, USA). *Miscanthus* was washed with deionized water and dried in a convection oven at 40 °C for 2 days. Washed and dried *Miscanthus* was ground using a Perten Grinder (Model No: 3600, Huddinge, Sweden). The particle size distribution test was performed using a set of 0.3, 0.5, 0.85, 2.0, and 2.36 mm sized sieves. In this study, the fraction with the particle size of ≤0.85 mm was used in the pyrolysis experiments.

### 2.2 Proximate Analysis

Proximate analysis of *Miscanthus* was carried out at Soil Lab, Biosystem and Agricultural Engineering, Oklahoma State University (Stillwater, OK, USA). Chemical composition of the biomass was carried out at Kansas State University (Manhattan, KS, USA). The total ash, carbon, nitrogen, dry matter, and mineral content of the samples were analyzed according to the standard methods of the AOAC^30^ and Soil Science Society of America.^31,32^

### 2.3 Preparation of Activated Carbon

Pyrolysis of *Miscanthus* was performed with and without chemical activation. In the chemical activation experiments, the biomass:chemical agent ratio (w/w) was chosen as 1:2 and 1:4, and ZnCl_2_ aqueous solutions were prepared according to the selected ratio. Then, the corresponding amount of biomass was added into aqueous ZnCl_2_ solution, and the mixture was continuously stirred using a magnetic stirrer (VWR, Atlanta, GA, USA) at room temperature (22 °C) for 24 h. Finally, obtained slurry was dried in a convection oven at 110 °C for 4 days until constant weight.

### 2.4 Experimental Design

The experimental design was set up according to the Box–Behnken design of surface response method using Design Expert software (version 12, StatEase, Mineapolis, MN, USA). Temperature, residence time, and activation ratio (biomass:chemical ratio) were the independent variables. Three different biomass:chemical agent ratios chosen for the design were: “0”, “–1”, and “1” representing no activation, 1:2 and 1:4 biomass: chemical agent (w/w), respectively. Process variables and their levels used in the experimental design are shown in Table 1. Two responses were selected as specific surface area (m^2^/g) and total pore volume (cm^3^/g). The pyrolysis experiments were carried out using an Across International STF1200 700 mm length tube furnace (Livingston, LJ, USA). All experiments were performed under 150 mL of N_2_ flow rate at a 20 °C/min heating rate. The pyrolysis reactor was purged with N_2_ for 30 min prior to each experiment.

**Table 1:** Process Variables and Their Levels Used in the Experimental Design^a^

	levels		
factors	–1	0	1
temperature (°C)	450	600	750
resistance time (min)	30	60	90
activation ratio	–1	0	1

a The experimental design of activated carbon (−1 and 1 show the biomass:chemical agent ratio of 1:2 and 1:4, 0 shows the process without chemical addition).

All 15 runs were carried out according to the experimental design. Resulting carbon materials were washed with distilled water until neutral pH and then dried in an oven at 100 °C for 24 h. The yield of carbon materials was calculated as^33^
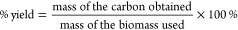
1

### 2.5 Characterization of the Carbon Material Produced

Morphology of the carbon samples was visualized using a scanning electron microscope (SEM) (Apreo 2, Thermo Fisher Scientific, Waltham, MA, USA). A Fourier transform infrared spectrophotometer (Nicolet iS50 FTIR, Thermo Fisher Scientific, Waltham, MA, USA) was used to determine the surface functional groups on the samples at a wavelength range of 600–4000 cm^–1^, 64 scans/s, and a spectral resolution of 4 cm^–1^. The BET (Brunauer–Emmett–Teller) surface area of the produced carbon was analyzed using N_2_ adsorption–desorption isotherms at −196 °C (Quantachrome Autosorb analyzer (Autosorb–1, Quantachrome Ins. Graz, Austria). The nitrogen adsorption data used to calculate the total pore volume (*V*_total_) were collected at a relative pressure, *P*/*P*o, of 0.995.^34^ The crystal structures of the carbon samples were examined by X-ray diffraction (XRD, Ultima IV, Rigaku, Tokyo, Japan). The XRD measurements were obtained at room temperature with a step of 6° in a range between 3 and 90°.

## 3 Results and Discussion

### 3.1 Proximate Composition of *Miscanthus*

Total nitrogen (TN), total carbon (TC), dry matter, acid detergent fiber (ADF), neutral detergent fiber (NDF), ash, and inorganic material contents of *Miscanthus* biomass used in this study are shown in Tables 2–4.

**Table 2:** Proximate Composition of *Miscanthus* (%, w/w)

TN	TC	dry matter	ADF	NDF	ASH
0.20	46.30	95.57	66.64	85.24	1.91

**Table 3:** Mineral Content of *Miscanthus*

P	Ca	K	Mg	S	Fe	Zn	Cu	Mn
%	%	%	%	%	ppm	ppm	ppm	ppm
0.061	0.118	0.248	0.045	0.029	79.8	21.1	6.0	168.3

**Table 4:** Lignocellulosic Composition of *Miscanthus*^a^

moisture (%)	glucan (%, db)	xylan (%, db)	arabinan (%, db)	lignin (%, db)	extractives (%, db)
3.65	41.03	17.75	2.08	20	13.58

a db: dry based

ADF and NDF indicate lignocellulosic components of the material. Hemicellulose content of the lignocellulosic material is calculated as 18.6% based on NDF and ADF contents.^35^ Detailed compositional analyses of the biomass are shown in Table 4. Mineral composition of the biomass *Miscanthus* is shown in Table 3.

Although the chemical composition of *Miscanthus giganteus* varies with the agronomic practices used during growth, harvesting time, soil type, and climate at the growth location, cellulose, hemicellulose, and lignin are the major components.^36,37^Table 4 shows that the *Miscanthus* precursor consisted mainly of cellulosic components (41.03%), followed by lignin (20%) and hemicellulose (19.93%). The extractives accounted for 13.58%, whereas the mineral content exhibited a low amount. The amount of cellulose and lignin is comparable with the study carried out by Pniewski et al.,^38^ however differing to the data obtained by Rutkowski et al.^39^ due to different practices as above.

### 3.2 Carbon Yield

The yields of activate d and nonactivated carbons from each experiment calculated according to eq 1 are given in Table 5.

**Table 5:** Yields of Activated and Nonactivated Carbon Materials

run	yield (%)	run	yield (%)	run	yield (%)
1	19.52	6	27.87	11	22.95
2	22.95	7	13.55	12	7.10
3	15.93	8	21.31	13	16
4	15	9	9.16	14	22.95
5	26.23	10	22.95	15	11.55

The carbon yields ranged from 7.10 to 27.87%. The experimental runs carried out in the presence of ZnCl_2_ produced significantly lower carbon yields than those runs without an activation reagent. In the case of all activated carbons, including Run2, Run5, Run6, Run8, Run10, Run11, and Run14, all samples exhibited a higher yield (%). In contrast to producing without chemical agents, activated carbon produced by chemical activation demonstrated lower carbon yields within combination of temperature, resistance time, and applied chemical agent ratio. In the case of ZnCl_2_ that was also concluded in the previous reports, an increase in the biomass:ZnCl_2_ ratio has resulted in a decrease in the carbon yield. The latter results can be explained by the carbon loss in the gas phase due to the formation of CO, CO_2_, and CH_4_ in the presence of ZnCl_2_ in the system.^23,40^ As seen Table 5, run 3 (600 °C, 90, −1) and run 15 (600 °C, 90, 1) have the same temperature and resistance time while using different chemical agent ratios effected on the carbon yield and led to lower the carbon yield for run 15 compared to run 3. The effect of temperature on the yield was also significant; higher pyrolysis temperatures resulted in lower carbon yield than the runs at lower temperatures even if the other process parameters was the same that can be concluded from run 7 (450 °C, 60, 1) and run 9 (750 °C, 60, 1). The other research group has reported similar results.^41^ In a previous study on pyrolysis of corn cobs in the presence of ZnCl_2_, we have also shown that carbon yield at 800 °C was lower than that at 500 °C.^42^ The other important factor affecting carbon yield is the type of chemical agent used for biomass impregnation. ZnCl_2_ and H_3_PO_4_ are shown to result in higher carbon yields than that obtained with KOH activation due to their effect on biomass decomposition efficiency and tar formation.^43^

### 3.3 RSM of the Pyrolysis Process Parameters

The experimental surface area and total volume data (Table 6) were statistically evaluated by the analysis of variance (ANOVA) method.

**Table 6:** BET and Total Pore Volume of Obtained Activated Carbon

run	factor 1: A: temp. (°C)	factor 2: B: time (min)	factor 3: C: biomass:ZnCl_2_ ratio	response 1: BET area (m^2^/g)	response 2: total pore volume (cm^3^/g)
1	450	60	–1	1371	0.796
2	750	30	0	124.4	0.071
3	600	90	–1	1428	0.933
4	600	30	–1	1788	1.053
5	450	90	0	0.423	0.002
6	450	30	0	0.001	0.001
7	450	60	1	678.8	0.440
8	750	90	0	246.7	0.141
9	750	60	1	1449	1.312
10	600	60	0	273.8	0.160
11	600	60	0	325.7	0.189
12	750	60	-1	1431	0.883
13	600	30	1	550.1	0.560
14	600	60	0	271.6	0.160
15	600	90	1	1121	1.164

RSM method is commonly used to examine the correlations among process variables.^27^ When experimental data do not fit a normal distribution, the “square root power transformation” method is used to normalize the skewed distribution and focus on visualizing certain parts of the data that are important for the study. “Square root power transformation” was applied to the BET data collected in this study because of the skewed distribution.^44^Figure S1 shows the ANOVA for response 1, BET surface area.

The model generated by the statistical evaluation was significant for response 1, and *A*, *C*, *A*^2^, *B*^2^, and *C*^2^ were the significant terms in the model, *p* < 0.05. The high coefficient of determination (*R*^2^ = 0.9839) for the quadratic model indicates that the model fits the experimental data well.^45^ The empirical formula for the response 1 is shown in eq 2.
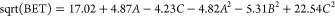
2

The model generated for response 2, pore volume, was also significant, and *A*, *B*, *AC*, *BC*, *A*^2^, *B*^2^, and *C*^2^ were the significant terms in the model, *p* < 0.05. The high coefficient of determination (*R*^2^ = 0.9971) for the quadratic model for response 2 indicates that the model fits the experimental data well. Table S2 shows the ANOVA results for response 2 (total pore volume). The empirical formula for response 2 (total pore volume) is shown in eq 3.
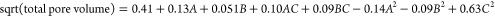
3

The interaction between variables of “temperature–time”, “temperature–activation ratio”, and “time–activation ratio” are shown as *AB*, *AC*, and *BC*, respectively. According to the ANOVA results, the *p*-value higher than 0.05 does not suggest a significant interaction between the variables for response 1. For response 2, *AC* and *BC* interactions were significant, indicating an available trend between the variables.

**Figure 1:**
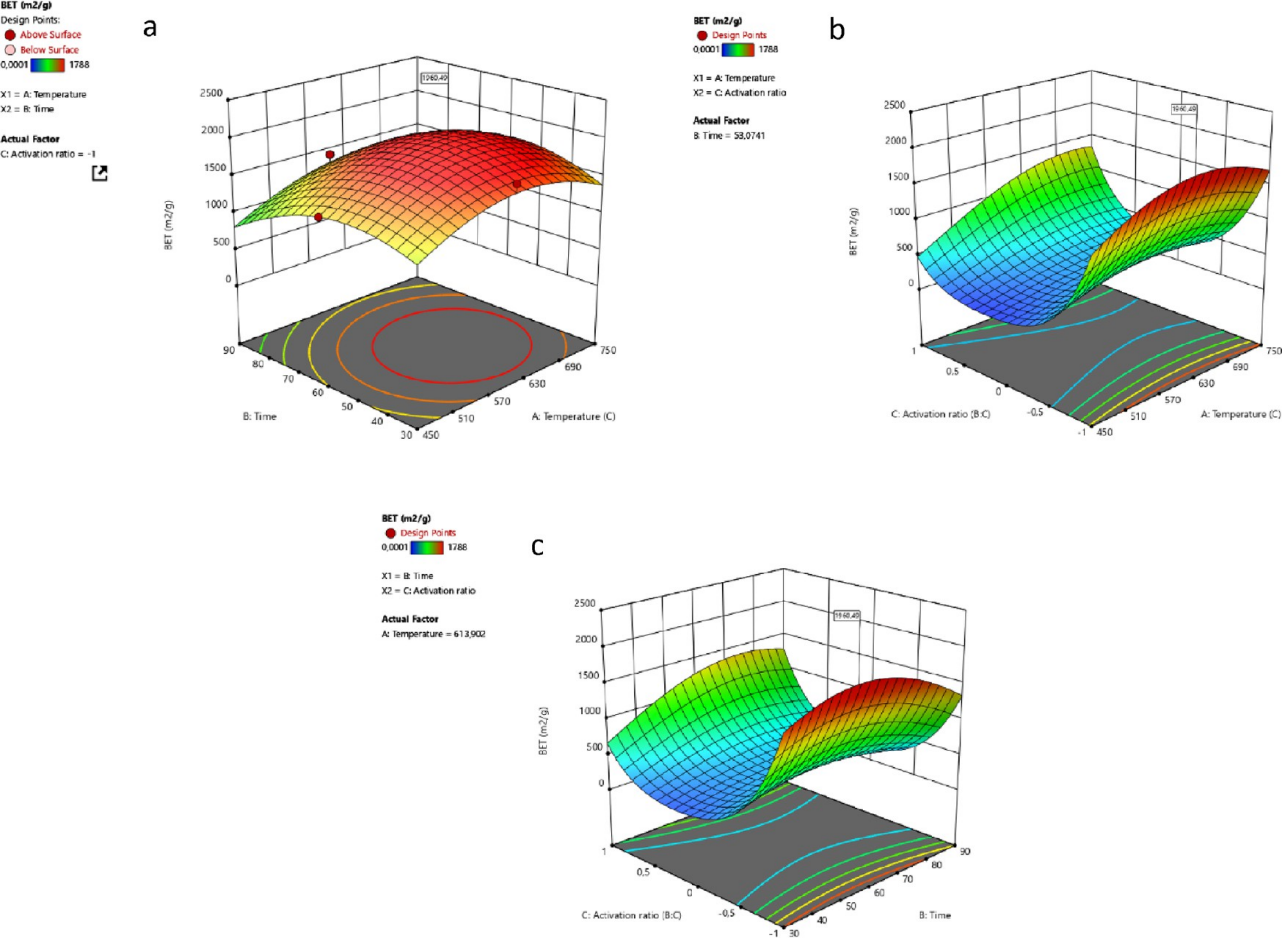
Interaction between (a) *AB* (temperature–time), (b) *AC* (temperature–activation ratio), and (c) *BC* (time–activation ratio) on response 1.

In experiment optimization, while the temperature, resistance time, and activation rate were determined as “in range”, responses were maximized. Carbon materials have to possess some properties such as high SSA, high pore volume, good electrical conductivity, and thermal conductivity to be used as energy storage material. Hence, the response values are maximized to determine the best condition that produces a carbon material with high surface area and porous volume desired for utilization in energy storage technologies. In line with the determined conditions, 64 different solutions were found. Among these, the condition providing the highest surface area and the condition providing the highest pore volume were chosen as two optimum conditions. The process conditions resulting in the highest surface area for response 1 were 614 °C, 53 min, and −1. The effects of interactions between different variables (*A*, *B*, *C*) on response 1 in line with the selected condition that is referred as OC1 are shown in Figure 1.

Figure 1a shows that the surface area increases with increasing temperature from 450 to 600 °C, but a significant decrease in surface area is observed at higher temperatures, over 614 °C. A similar trend is observed for the residence time. Over 30 min, the surface area increases with increasing temperature, reaching its highest level at 53 min. When increased further, the lowest surface area is observed. The interaction of temperature and activation rate (AC) is significant (*p* = 0.0014), and the surface area increases with increasing temperature as the activation rate goes from 0 to −1. However, the further increase in the activation ratio and temperature causes a decrease in the surface area. The time and activation ratio (*BC*) interaction indicates that the BET surface area increases as the activation rate goes from 0 to −1 and reaches its highest level when the time is increased up to 53 min. Under the optimum conditions, the BET surface area is expected to be 1951.8 m^2^/g and the total pore volume to be 1.169 cm^3^/g with a desirability of 0.971.

**Figure 2:**
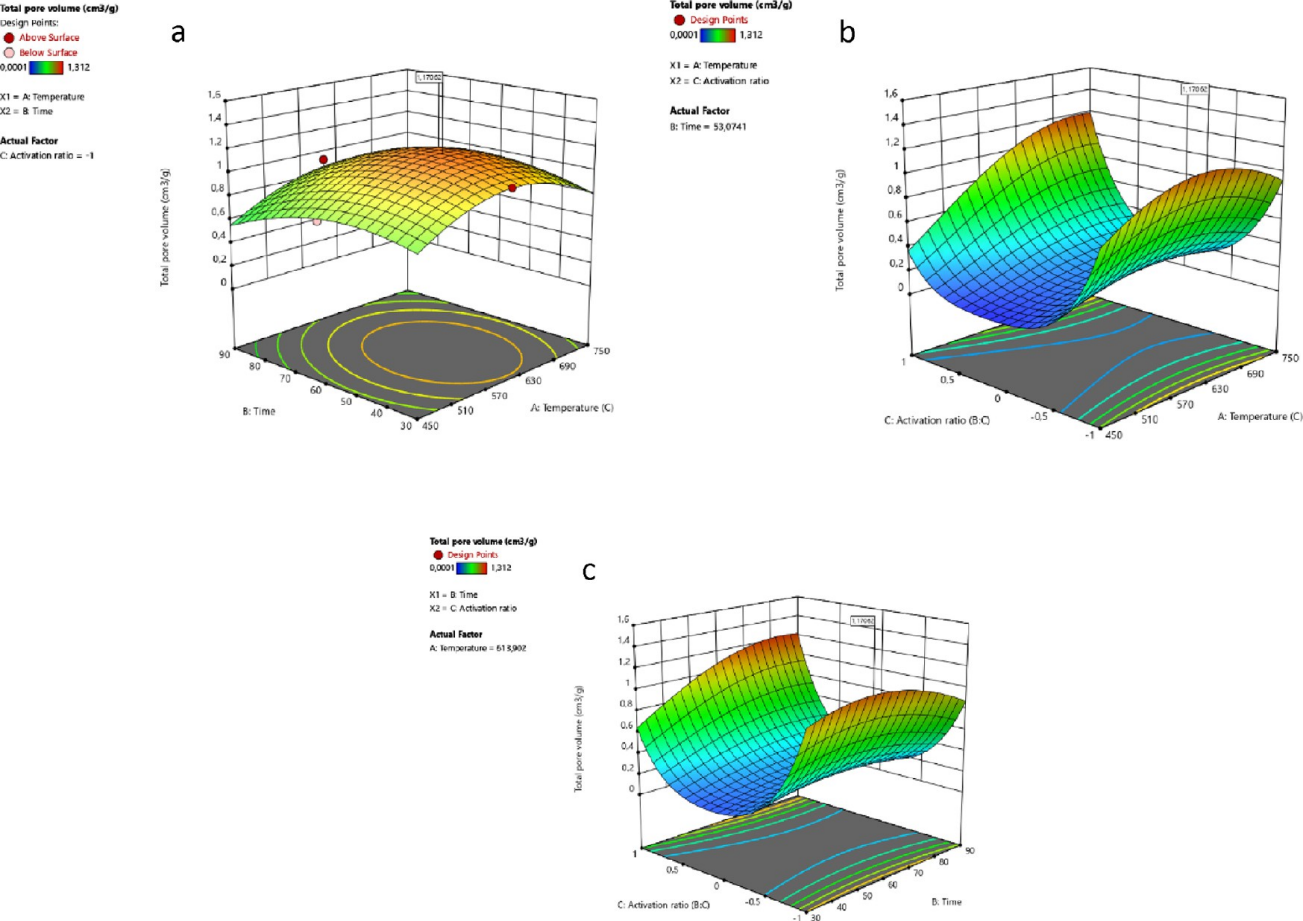
(a) Interaction between *AB* (temperature–time), (b) *AC* (temperature–activation ratio), and (c) *BC* (time–activation ratio) on response 2.

Figure 2 shows the effects of temperature, time, and activation ratio on pore volume, response 2. The pore volume increases as the temperature increases from 450 °C, and time increases from 30 min until it reaches to its maximum at 722 °C and 77 min. The optimum activation ratio at the latter conditions (Figure 2) shows the negative effect on the pore volume with further increase. It is seen that the activation rate is effective in obtaining a high pore volume for chemically activated carbons, with the effect of temperature (*AC*). When the activation rates are examined, a total pore volume of 1.170 cm^3^/g can be obtained at 614 °C, by using the 1:2 biomass:chemical ratio. The same pore volume can be achieved at a 1:4 ratio when the temperature exceeds 700 °C. When the time–activation ratio (*BC*) interaction is examined, it is seen that an increase in the biomass:chemical ratio will result in an increase in temperature and resistance time, as in the *AC* interaction.

**Figure 3:**
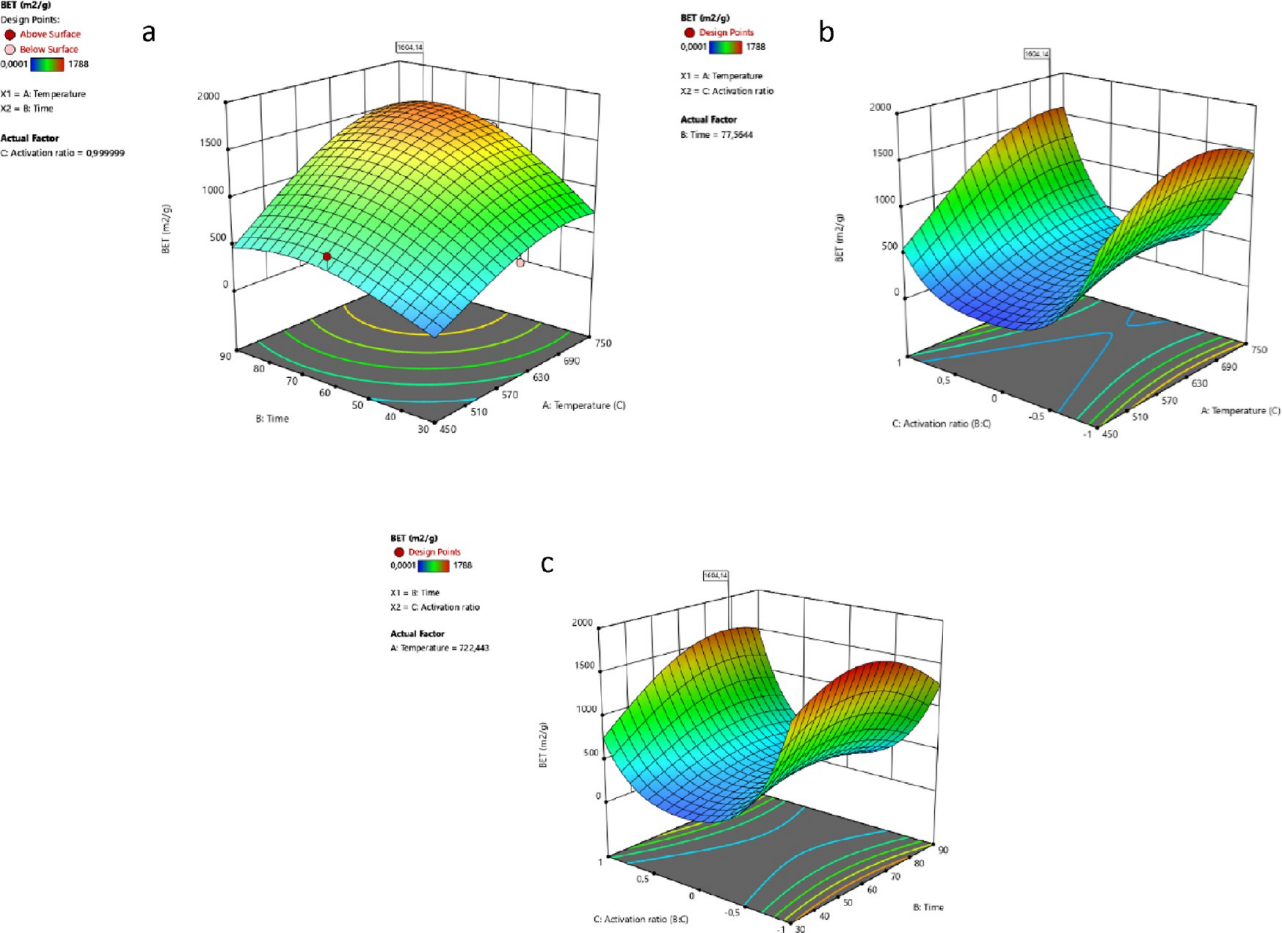
(a) Interaction between *AB* (temperature–time), (b) *AC* (temperature–activation ratio), and (c) *BC* (time–activation ratio) on response 1.

The second optimum conditions for the highest total pore volume were determined as 722 °C, 77 min, and 1 (1:4 biomass:chemical) activation ratio. Under the optimum conditions, 1,595.4 m^2^/g surface area and 1.439 cm^3^/g total pore volume can be obtained. Figure 3 shows the effects of the selected conditions on the BET surface area.

**Figure 4:**
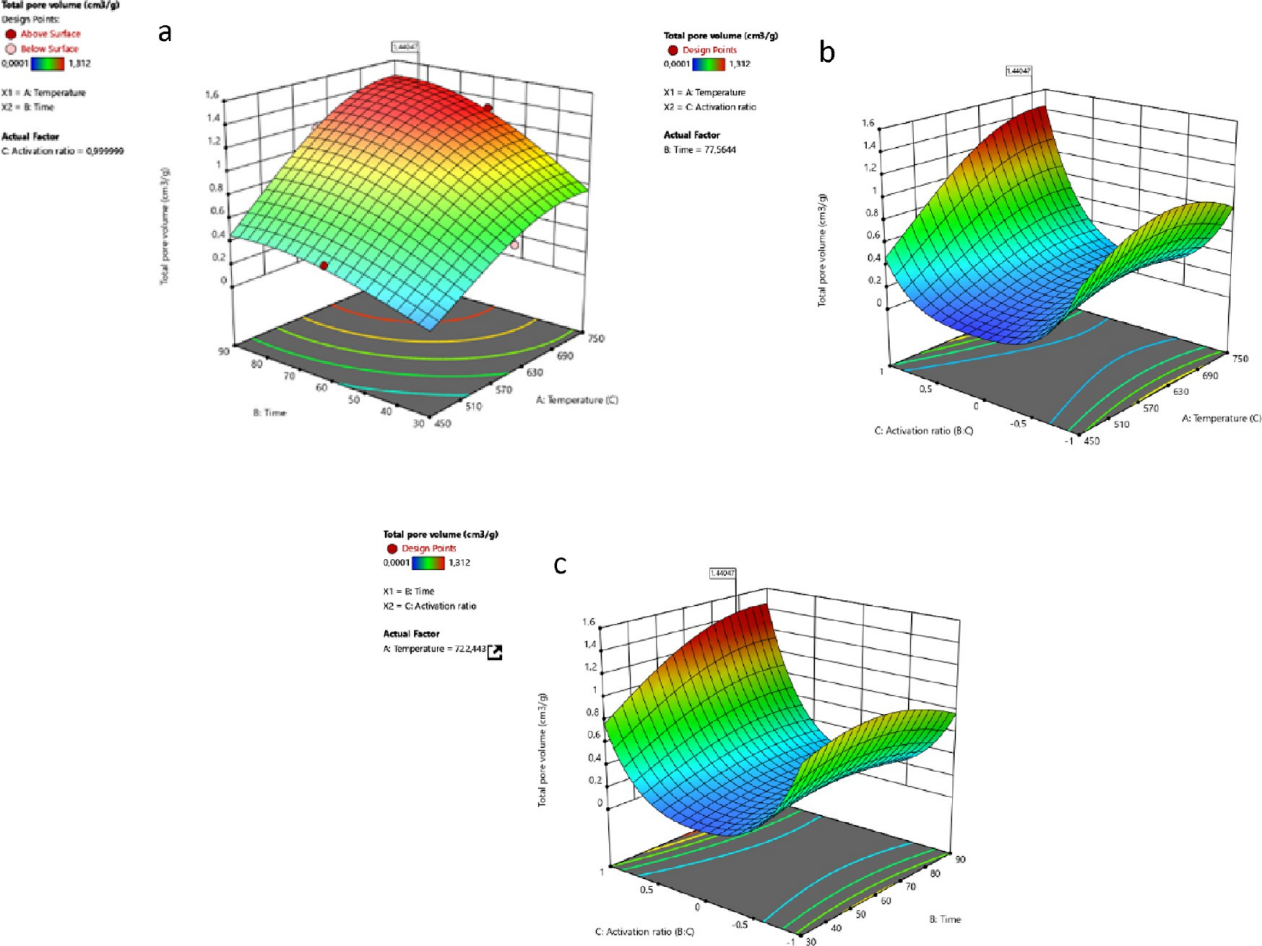
Interaction between (a) *AB* (temperature–time), (b) *AC* (temperature–activation ratio), and (c) *BC* (time–activation ratio) on response 2.

When the *AB* (temperature–time) interaction for the second condition is examined, it is seen that high surface area can be obtained at high temperatures and longer resistance times at a constant activation rate of 0.99. However, it can be seen from the data in Figure 3a that the most important parameter in the *AB* interaction is the temperature value and that high surface area cannot be reached at a constant activation rate (0.99) in case the temperature is low (450 °C) during long resistance times (90 min). When *AC* (temperature–activation ratio) is examined, it is seen that the highest BET value can be reached at high temperatures and at a biomass:chemical ratio of 1:4 (1) in a fixed time (77 min). When the *BC* (time–activation ratio) pair is examined, it is seen that the highest BET value can be reached as the resistance time and activation ratio (1) increase. Figure 4 shows the effect of the second optimum condition on the total pore volume.

When the *AB* (temperature–time) relationship is examined for the second condition, the increase in temperature and resistance time at a constant activation rate (0.99) results in an increase in the total pore volume. A similar trend is seen in the *AC* (temperature–activation ratio) pair. The increase in temperature and activation at a fixed resistance time (77 min) results in an increase in the total pore volume. When the *BC* (time–activation ratio) pair is examined, the increase in time and activation rate at constant temperature (722 °C) increases the total pore volume.

### 3.4 Characterization of Activated Carbon

According to the nitrogen adsorption–desorption isotherm, the highest BET surface area are in following order: run 4 (600 °C, 30 min, −1) > run 9 (750 °C, 60 min, 1) > run 12 (750 °C, 60 min, −1) > run 3 (600 °C, 90 min, −1) > run1 (450 °C, 60 min, −1). Among these runs, run 4, run 9, and run 12 were chosen due to show the highest BET area and varied pore volume. Average pore diameters of run 4, run 9, and run 12 are 2.2, 3.6, and 2.4 nm, respectively. Temperature is a significant effect on pore diameter that leads to collapse on the pore walls and increases the average pore diameter.^46^ Even if run 9 and run 12 are carried out at the same temperature (750 °C), different biomass-to-chemical agent ratios effect their average diameter.

**Figure 5:**
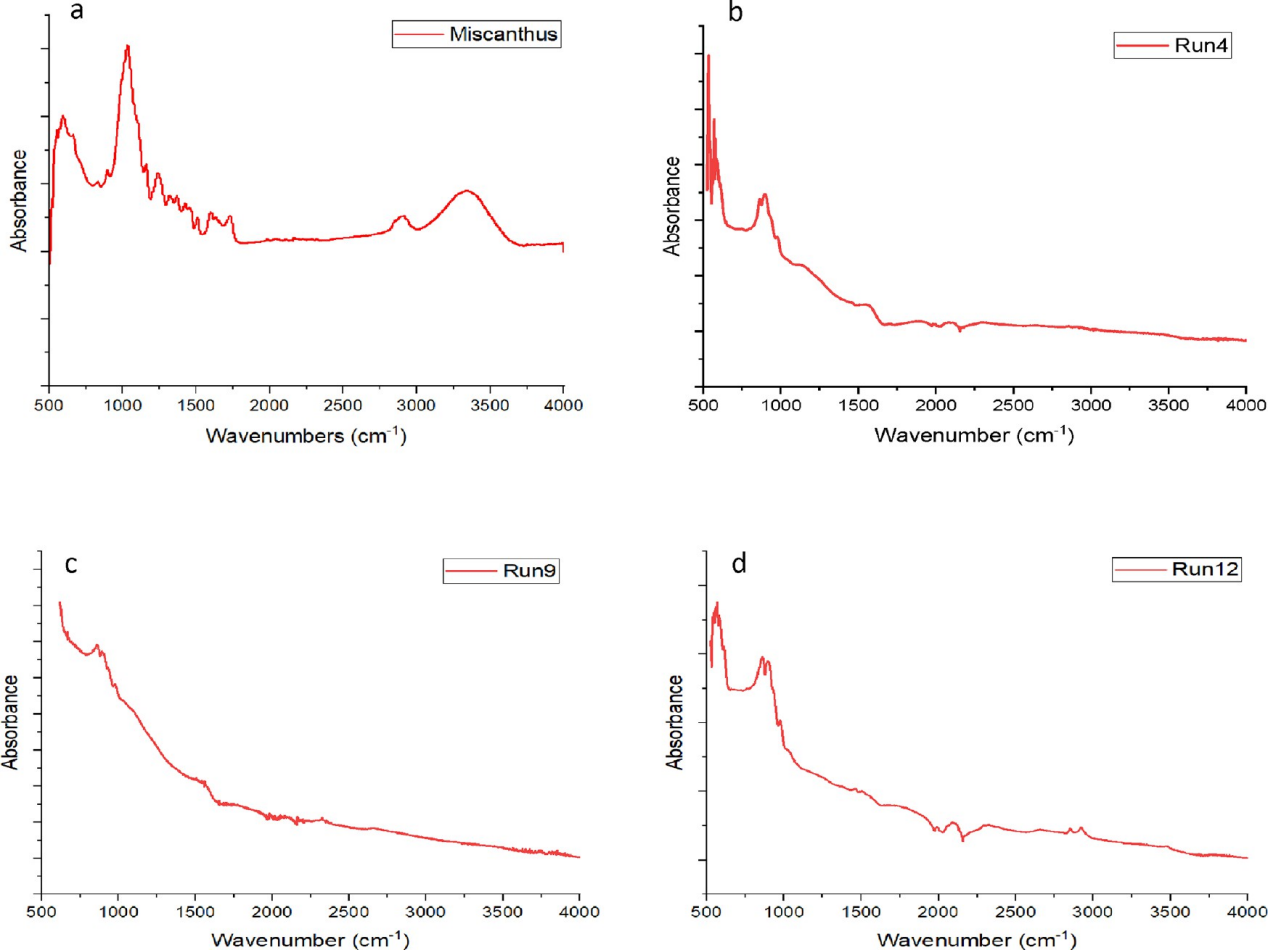
FTIR spectrum of (a) *Miscanthus*, (b) run 4, (c) run 9, and (d) run 12.

In order to identify chemical and morphological properties, FTIR, XRD, and SEM analyses were carried out on the carbon samples produced. FTIR allows identification of the functional groups present in the material analyzed.^47^Figure 5 shows the FTIR spectrum of *Miscanthus* (a), run 4 (b), run 9 (c), and run 12 (d). As seen in Figure 5, increasing temperature resulted in a decrease in the intensity of bands for all runs compared to unprocessed *Miscanthus*. *Miscanthus* has one broad band between 3000 and 3250 cm^–1^ and a weak band between 2750 and 3000 cm^–1^ that are related to O–H stretching vibration. The C–H peak at 2840–2950 cm^–1^ was seen only in the FTIR spectrum for the raw *Miscanthus*. Due to the decrease in the polarity of material and oxygen loss, peaks in the other sample showed less intensity.^48^ Raw *Miscanthus* showed a weak peak around 1750 and 1650 cm^–1^ and a sharp peak at 1000 cm^–1^. As seen Figure 5a, the peak around 1740 cm^–1^, which represents the nonconjugated C=O bond in hemicellulose stretching vibration,^49^ in raw *Miscanthus*, was missing in all other samples due to the biomass degradation at high temperature. Similar results were reported by Grams et al.^47^ They observed a decrease in the intensity of bands.^47^

**Figure 6:**
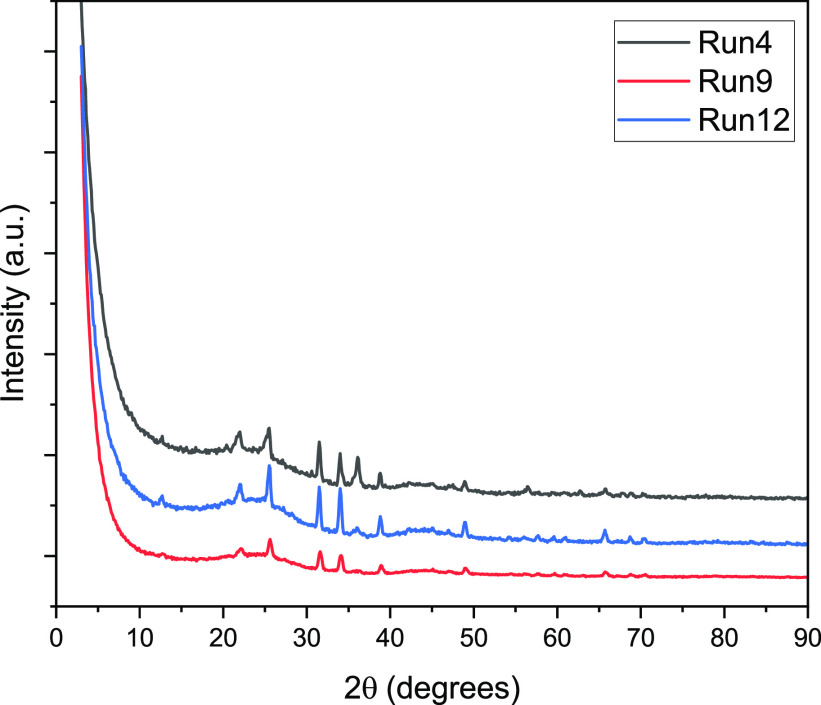
XRD results of (e) run 4, run 9, and run 12.

The crystal phase of the activated carbons was confirmed by XRD. The pattern of the samples is shown in Figure 6. The run 4 sample has one diffraction peak around 26°,which belongs to the (002) crystal plane and several other small diffraction peaks around 35, 38, 46, 48, and 62°. The small intensity peak around 2θ = 26° refers to the typical (002) crystal plane of graphite.^50^ The diffraction peaks near 2θ = 26° are also present in run 9 and run 12. However, run 12 showed high diffraction peaks different than the other two samples.

**Figure 7:**
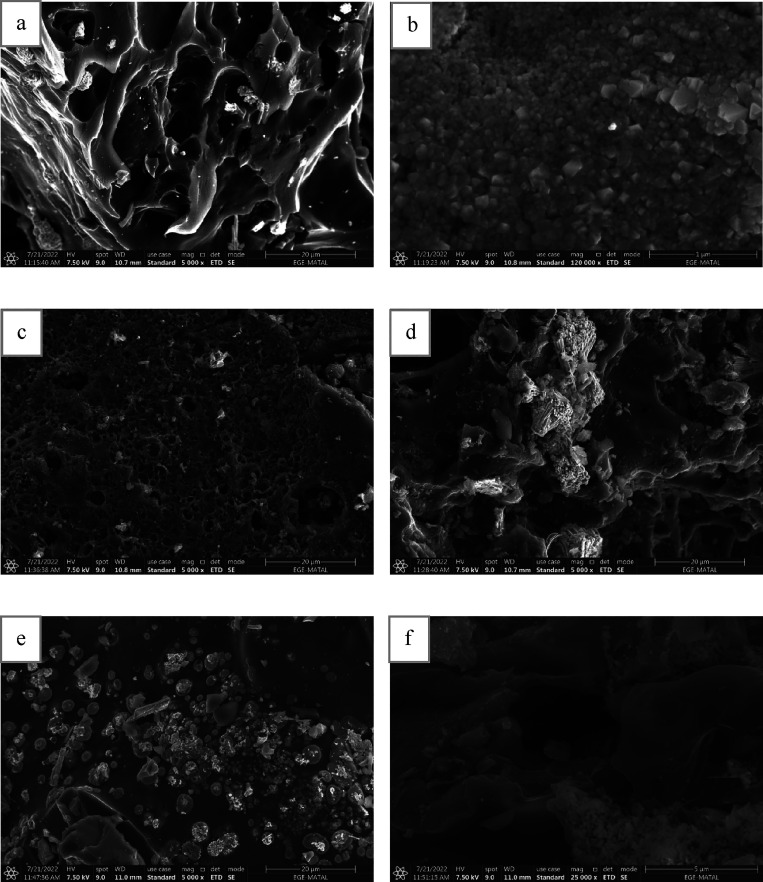
SEM analysis of (a) run 4 (5000×), (b) run 4 (120,000×), (c) run 9 (5000×), (d) run 9 (5000×), (e) run 12 (5000×), and (f) run 12 (25,000×).

The surface morphology and porous structures of activated carbon derived from *Miscanthus* are shown in SEM images in Figure 7a–f, which show surfaces and porous structure of run 4, run 9, and run 12. The sample show abundant sphere-like particles. The sample consisted of nanosized particles gathered in an interconnected porous framework. Forming pore channel structures and the highest surface area through ZnCl_2_ activation is due to these spheres that produce plenty of gaps (Figure 7b). Compared to run 4, run 9 and run 12 consisted of nonuniform structure. Due to high pyrolysis temperature and resistance time, a large particle occurred on the surface.

### 3.5 Characterization of the Carbon Produced at Optimum Conditions

BET analysis were carried out for activated carbons obtained at optimum conditions (OC1 and OC2), Table 7.

**Table 7:** BET and Total Pore Volume of OC1 and OC2

sample	*S*_BET_ (m^2^/g)	*V*_T_ (cm^3^/g)	*S*_mic_ (m^2^/g)	*V*_micro_ (cm^3^/g)	*S*_meso_ (m^2^/g)	*V*_meso_ (cm^3^/g)	*D*_av_ (Å)
OC1	1415.4	0.748	774.91	0.375	640.49	0.373	21.13
OC2	1499.8	1.443	10.98	0.010	1488.8	1442.9	38.47

**Figure 8:**
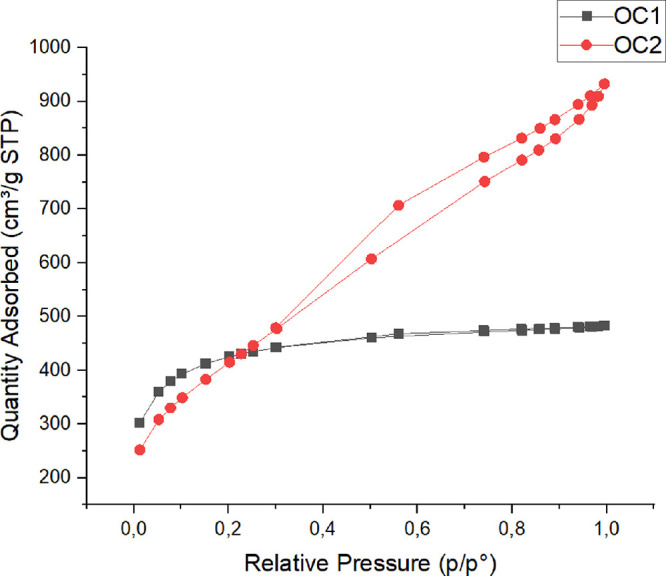
N_2_ adsorption–desorption isotherms of response 1 and response 2.

The experimental surface area was similar to the area predicted by the OC2 model. However, the experimental surface area was found to be smaller than the area predicted by the OC1 model, yet the carbon material had a predominantly microporous structure. In Table 7, the mesopore surface area (S_meso_) and mesopore volume (*V*_meso_) were calculated by subtracting *S*_mic_ from *S*_BET_ and *V*_micro_ from *V*_T_, respectively.^51^Figure 8 shows the N_2_ adsorption–desorption isotherms of OC1 and OC2.

Based on the IUPAC classifications of physiosorption isotherms, OC1 showed I-type isotherms that indicate a microporous structure.^52^ However, OC2 exhibited IV-type isotherms that show condensation in meso-/macropores with hysteresis loops at the relative pressure of *P*/*P*o = 0.4.^46^ Similarly, a micro-/mesoporous carbon structure was obtained with elephant grass activated with ZnCl_2_.^53^ ZnCl_2_ is considered a good activator for producing carbon material with a microporous structure.^54^ However, optimization of the biomass-to-ZnCl_2_ ratio is crucial for achieving a desired pore structure in the final product. At a low biomass/ZnCl_2_ ratio, a microporous carbon structure is produced, while higher ratios lead to the collapse of the microstructure creating a carbon material with a mesoporous structure.^53,55,56^

Specific surface area, distribution of pore-size, and electrical conductivity are critical properties for choosing a carbon material to be used in electrochemical energy storage applications. For instance, the presence of macropores in the material provides a fast electron-transfer path for ion transport, while micro- and mesopores create high SSA.^57^ Among the porous structure, mesopore volume with high percentage in the hierarchical pore structure of activated carbon plays a key role for obtaining high capacitance and also rate performance in the electrolyte.^58^ A carbon material having a pore structure consisting of mostly micro- and mesopores is desirable for shape stabilization of PCM to be used in TES. Micro- and mesoporous structures that have small pores and large surface areas prevent the leakage problem of PCMs.^59^ Owing to surface tension and capillary forces, micropores and mesopores are the most suitable kinds of nanoscale structures.^45^ The carbon material produced under the conditions determined by the OC1 model had mostly micropores (774.9 m^2^/g) and mesopores (640.4 m^2^/g) that are suitable as supporting material for PCM applications.^60^

Intensity of the peaks found in the FTIR spectra of the materials produced under the process conditions determined by both OC1 and OC2 models were weaker than those of the unprocessed biomass, Figure S3a,b. The latter result can be explained by the effect of carbonization and activation on the functional groups in the material. Similar results were obtained by Li et al. for carbonized material having a weaker peak than original material.^61^ OC1 (Figure S3a) showed a weak peak at 3000 cm^–1^ related to O–H stretching, while OC2 (Figure S3b) showed an intense peak at the same wavelength. OC1 has a small peak at 2350 and 2000 cm^–1^, which are attributed to the O=C=O stretching and C=C=N stretching vibrations, respectively. OC1 also have a peak at 1550 cm^–1^ that belongs to N–O stretching.^62^ The peak at 1150 cm^–1^ is attributed to the C–O stretching. OC1 showed a broad band from 500 to 1000 cm^–1^ that is formed by C–O stretching.^63^

Surface functional groups on carbon have significant effects on the efficiency of electrochemical capacitors.^59,64^ Similar to OC1, OC2 has the same peak at the same wavelength but with a higher intensity. Both OC1 and OC2 have abundant oxygen functional groups. OH groups on AC can serve a hydrophilic surface to the ACs, thus easing ion transport in their nanosize pores.^65^

## 4 Conclusions

Activated carbon with a high BET surface area and total pore volume was produced from *Miscanthus*, using ZnCl_2_ as the activating agent. Box–Behnken design of the surface response method was used to determine the effects of process parameters on BET surface area and total pore volume. Different optimum conditions having separately the highest surface area and pore volume were obtained by optimization study in order to examine how the surface area and porous structure of activated carbon affect TES and EES. Based on the optimization study, two different pyrolysis process conditions, 614 °C, 53 min, and −1, and 722 °C, 77 min, and 1, gave the BET surface area of 1415.4 m^2^/g and 0.748 cm^3^/g total pore volume for optimum condition 1 (OC1) and 1499.8 m^2^/g the highest BET surface area and 1.443 cm^3^/g total pore volume for optimum condition 2 (OC2), respectively. Activated carbon that was obtained from OC1 showed highly microporous and mesoporous structures, while OC2 exhibited mesoporous and macroporous structures. Both OC1 and OC2 showed an oxygen-rich functional group. Owing to their porous surface properties, activated carbon obtained from OC1 is a good candidate as support material for PCMs in thermal energy storage thanks to its micropore and mesopore structure that resulted in preventing the leakage problem of shape-stabilized PCM. On the other hand, OC2 activated carbon is suitable as electrode material in electrochemical energy storage devices due to serving as a hydrophilic surface to AC. Hence, ion transport in its interconnected pore structure is enhanced. Optimizing the selection of energy storage systems in compliance with SDG (Sustainable Development Goals) is crucial to maintain efficient production strategies. Using bio-based materials in energy storage serves responsible consumption and production goals, while characterization attempts as targeted in this study is believed to help innovate existing methods for the utilization of different materials and accurate assessment of energy storage technologies.

## Supporting Information Available

Activated carbon, chemical agents, process conditions, BET area, BJH, and Figures S1–S3 (PDF)
