# Supplementary material for: Miscanthus-Derived Energy Storage System Material Production
Source: ACS Omega. 2023 Feb 22;8(9):8779–90. doi: 10.1021/acsomega.3c00024 (PMC9996796; doi:10.1021/acsomega.3c00024)
Supplement: Supplementary file 1 — ao3c00024_si_001.pdf [file ao3c00024_si_001.pdf]

Supporting Information

MISCANTHUS DERIVED ENERGY STORAGE SYSTEM MATERIAL PRODUCTION

Fikret Muge Alptekin[a,b], Nurhan Turgut Dunford[b] , Melih Soner Celiktas[a*]

[a] Ege University, Solar Energy Institute, 35040, Izmir, Turkey

[b] Oklahoma State University, Department of Biosystems and Agricultural Engineering, Stillwater, OK, USA

*soner.celiktas@ege.edu.tr, nurhan.dunford@okstate.edu, *f.mugealptekin@gmail.com

Table S1. Activated carbon derived from different biomass, chemical agent and process conditions

**Biomass**	**Condition**	**Chemical agent/s**	**Preparation**	**Washing**	**BET**	**BJH**	Ref
Coffee grounds	One-pot synthesis<br><br>700 C for 2 h in N_2 (there is no data regarding flow rate of N_2)	KOH, NaOH, HCl, H_3PO_4, $ZnCl_2$ and $FeCl_3$	Wet impregnation	1 M HCL to remove the metallic potassium and potassium compounds	The highest BET was achieved with $ZnCl_2$ (1242 m^2/g) and KOH (1250 m^2/g)	For $ZnCl_2$ and KOH, achieved Vtotal 0.321 and 0.167 cm^3/g, respectively.	1
Albizia procera leaves	850 C for 5 h under N2 Heating rate was 10 C/min	$NaHCO_3$, $ZnCl_2$ and without chemical agent	Mass ratio of biomass: $NaHCO_3$ 1:2 Mass ratio of biomass:$ZnCl_2$: 1:4	Washed with in an aqueous solution of 1 M HCl by ultrasonification and subsequent centrifugation<br>-then washed 3 times with deionized water<br>-dried 60 C for 24 h	The highest BET was achieved with $NaHCO_3$ as 910 m^2/g<br><br>SSA of activated with $ZnCl_2$ and without chemical agent were 777 m^2/g and 322 m^2/g	Vtotal information is not available<br><br>However, activated with $NaHCO_3$ showed higher micro/mesopore ratio<br><br>$ZnCl_2$ showed mixing of micro and mesoporous structure	2

Pecan nutshell	Solar pyrolysis<br><br>One-step carbonization/chemical activation<br><br>Before pyrolysis, pecan nutshell was treated with hot water at 80 C for 10 min.<br>As a control, untreated pecan nutshell was also used for pyrolysis.<br><br>800 C for 1 h with an averaged heating rate of 14.5 °C min−1	H_3PO_4	Biomass:H_3PO_4<br>1:2<br><br>Wet impregnation with concentrated H_3PO_4	Carbon materials rinsed in a Soxhlet reflux condenser with deionized water until pH was reached neutral	SSA of untreated and treated carbon materials were 781 m^2/g and 1085 m^2/g, respectively.	Vtotal of untreated and treated carbon materials were 0.518 and 0.682 cm^3/g, respectively.	3
Walnut shell	Hydrothermal carbonization at 180 C for 12 h<br><br>Obtained carbon was treated with K_2CO_3 and carbonized varying temperature such as 600, 700 or 800 C for 2 h in a N_2 atmosphere	K_2CO_3	Wet impregnation<br><br>Biomass:K_2CO_3 ratio was 1:1	N.A	N.A	N.A	4
Corn stalks core (CSC)	2 stages carbonization CSC firstly carbonized at 400 C for 2 h under N_2 and obtained material impregnated with KOH	KOH	Biomass:KOH<br>1:3	Obtained activated materials purified in 2 M HCl within water bath at 90 C for 1 h and washed	The highest SSA obtained at 700 C as 2349.37 m^2/g<br><br>Original	0.6 cm^3/g pore volume	5

	solution.<br><br>For activation, impregnated carbon material was heated at 400 C for 30 min and temperature increase to 500-800 C for 1 h.			with deionized water until pH reach to neutral.<br><br>Dried at 60 C.	corn stalk core powder has SSA of 35.65 m^2/g		
Tea residue	Chemical activator and semicarbonized samples were calcined at 400 C for 1 h in N_2.	$ZnCl_2$, H_3PO_4, H_2SO_4, NaOH, and KOH<br><br>Different concentration solution (5, 30, 50 %)	Wet impregnation<br><br>Tea residue:chemical activator 1:6	The activated sample was soaked with a solution of 0.01 M hydrochloric acid and stirred 10 min and washed distilled water.<br><br>Dried at 110 C for 24 h.	The BET area of 1:7 immersed $ZnCl_2$ activated carbon was 871.03 m^2/g	Vtotal: 0.4221	[6]
miscanthus	Obtained mixture activated at 120 C for 3h, at 450 C for another 2h and 750 C for final 3 h under Ar.<br><br>To show effect of each chemical, different variations were used.	KOH, urea and ammonia solution	Wet impregnation<br><br>1 g of miscanthus were mixed with 20 ml of ammonia solution and stirred 24 h. then 4 g of KOH and 1 g of urea were added and stirred another 24 h	Ultrasonicated in 2M HCL solution for 1 h and washed with distilled water until pH reach to neutral<br><br>Dried at 70 C at least 12 h	The BET surface area of activated carbon that mixed all three chemical was 2359.1 m^2/g	0.99 cm^3/g	[7]
Waste potato peel	Biomass material that mixed with chemical agent and dried was activated in	KOH, $ZnCl_2$ or H_3PO_4	Wet impregnation	The obtained product washed with Soxhlet			[8]

	different temperature (400, 600 and 800 C) for 2 h			apparatus for 24 h until constant pH and then with ethanol Dried at 100 C for 24 h in a vacuum furnace			
Euphorbia rigida	3 different physical activation including static atmosphere, nitrogen and steam were applied at 550 C to obtain biochar.<br><br>Obtained biochar was substituted to the chemical activation with different chemical agent at 550 C at a rate of 10 C/min under either nitrogen or steam atmosphere.	HCl, KOH, K_2CO_3, H_2SO_4, H_3PO_4, NaOH, $ZnCl_2$	Wet impregnation<br><br>Biomasss:chemical agent 1:0.25, 1:0.5, 1:1<br><br>Char and liquid was stirred at 300 rpm for 2 h and then keep at room temp for 24 h.<br><br>Slurry dried at 105 C for 48 h.	Washed with hot distilled water until reaching a stable pH of around 7.	The highest BET was achieved with K_2CO_3 (biomass: K_2CO_3 1:1) as 1079 m^2/g	The micropore volume and total volume of 0.443 cm^3/g and 0.556 cm^3/g, respectively.	[9]
giant miscanthus, corn stalk, and wheat stalk	First, carbonization process was carried out at 800 C for 1 h under N_2 gas with a flow rate of 300 cm^3/min and heating rate of 3 C/min<br><br>The activation carried out with obtained carbon at 900 C for 1 h	KOH	Dry mix<br><br>Biomass: KOH 1:4	Obtained powders were washed with 0.1. M HCL solution and deionized water until reaching pH 7.<br><br>Dried at 80 C for 16 h.	The highest BET achieved with corn stalk derived activated carbon as 2434 m^2/g.<br><br>The BET of giant miscanth	Vtotal for corn stalk, giant miscanthus and wheat derived activated carbon were 1.22 cm^3/g, 0.99 cm^3/g and 1.10 cm^3/g.	[10]

	and a heating rate of 2 C/min under Ar atmosphere.				us and wheat derived activated carbon were 2212 m^2/g and 2327 m^2/g, respectively.		
Amygdalus pcdunculata shell	Chemically treated sample was carbonized at 350 C for 1 h and activated at desired varying temperature (750, 800, 850, 900 and 950 C) for 60, 90, 120 and 150 min under N_2 flow 0f 100 ml/min at a heating rate of 5 C/min	KOH	Wet impregnation<br><br>Biomass: KOH (1:0.5-3.5)	Obtained activated carbons were boiled in 0.1 M HCL for 5 min and rinsed repeatedly with distilled water to remove retained chemicals until the filtrate reached neutral pH.<br><br>Dried at 105 C for 24 h.	The max BET achieved as 2059 m^2/g	N.A.	11
Pine wood	Biomass firstly pyrolyzed at 360 C for 1 h.<br><br>Obtained product impregnated with chemicals with different varying biomass to chemical ratio for two chemicals were pyrolyzed at 580 C for 2 h.	$ZnCl_2$ and KOH	Wet impregnation<br><br>Biomass: $ZnCl_2$: 1:1 Biomass:KOH 1:4	Obtained activated carbon powder was poured into deionized water, and pH adjusted to 7 using 1.0 N HCl with vigorous stirring and recovered after	The highest BET was achieved with $ZnCl_2$ as 1,332.0 m2/g	Pore volume was 0.37 cm^3/g for activated carbon with $ZnCl_2$	12

				filtration and dried at 105 C			
pineapple waste biomass (leaves, stem, crown)	Pineapple waste biomass mixed with $ZnCl_2$ and immersed for 24 h at room temperature with occasional stirring. After drying, it carbonized at 500 C for 1 h.	$ZnCl_2$	Wet impregnation<br><br>Biomass: $ZnCl_2$ 1:0.5-1-1.5<br><br>Different ratio of $ZnCl_2$ was conducted to pineapple leaf due to the highest BET achieved with this material	The carbonized samples washed with warm distilled water (30-35 C) to remove residual $ZnCl_2$.<br><br>Dried at 100 C for 24 h.	The highest SSA achieved with pineapple leaf with 1002 m^2/g.	The highest Vtotal achieved with biomass: $ZnCl_2$ as 0.56 cm^3/g	13
Fox nutshell	Fox nutshell was treated with 0.5 N NaOH to remove impurities and washed distilled water<br><br>One step chemical activation process was carried out with different temperature (500 to 700 C) and a heating rate of 5 C/min for 60 min with N_2 flow of 150 ml/min	$ZnCl_2$	Wet impregnation<br><br>Different $ZnCl_2$ ratios of 1-2.5 to biomass	Obtained carbonization material was soaked with 0.5 N HCl solutions for 24 h. Soaked carbonized material was washed several times with hot distilled water and finally cold distilled water until the pH of wash water become neutral.<br><br>Dried at 110 C for 24 h.	The highest BET of 2869 m^2/g achieved with carbon material that was 2:1 impregrated and carbonized at 600 C.	The highest Vtotal of 1.96 cm^3/g achieved with carbon material that was 2:1 impregrated and carbonized at 600 C.	14

Cacao pod husk	Two stage carbonization; hydrothermal carbonization and activated carbon production<br><br>Hydrothermal carbonization was carried out different temperature range (200-225 C)<br><br>Activation carried out at 600 C for 1 h under N_2 flow.	$ZnCl_2$	Wet impregnation<br><br>Biomass: $ZnCl_2$ 1:4	The obtained activated carbon was washed using 0.1 M HCl solution and DDI water subsequently.<br><br>Dried at 105 C for 24 h.	The highest BET of 1694.91 m^2/g achieved with activated material that conducted hydrothermal carbonization at 225 C.	NA	15
Nori	Activation was carried out at two different temperature (700 or 800 C) with a heating rate of 5 C/min for 2 h under N^2 flow.	$ZnCl_2$	Mixing with $ZnCl_2$ and a little water<br><br>Nori: $ZnCl_2$ 1:1, 2:1, 4:1	Obtained carbons were washed with excessive 10% HCl and deionized water.	The max BET was achieved as 832.4 m^2/g with the sample that activated 1:1 at 700 C.<br><br>Increasing of biomass ratio resulted in decreasing of BET area.	Vtotal was 0.54 cm^3/g for the sample that activated 1:1 at 700 C.	16
cotton	$ZnCl_2$-impregmated cotton was carbonized at 850 C for 2 h under 60 ml/min N_2 flow	$ZnCl_2$	Wet impregnation for 6h<br><br>Biomass: $ZnCl_2$ 1:0, 1:1, 1:2, 1:3	The obtained carbon washed with 1 M HCl and DI water several times, and dried in air.	The highest BET area achieved as 1620.0 m^2/g with the sample that activated	Vtotal was 0.992 cm^3/g for the sample that activated 1:3.	17

				(it is not available in text, reached from referred ref.)	1:3.<br><br>Without $ZnCl_2$, activated carbon showed 89.6 m^2/g of BET surface area.		
Ramie fiber	Impregnated material was carbonized at 650 C at a heating rate of 5 C/min for 2 h.	$ZnCl_2$	Wet impregnation with $ZnCl_2$solution of different concentrations (10,20,40 and 60 %)	Obtained carbon materials were washed in 1 M HCl, rinsed in distilled water to neutral, and then filtered and dried.	The highest BET areas were achieved as 1893 and 1827 m^2/g with the samples that were activated with 40% and 60% concentration of $ZnCl_2$, respectively.	the highest Vtotals were achieved as 1.28 and 1.78 cm^3/g with the samples that were activated with 60% and 40% concentration of $ZnCl_2$, respectively.	18

ANOVA for Quadratic model

Response 1: BET
Transform: Square Root
Constant: 0

Source	Sum of Squares	df	Mean Square	F-value	p-value	
**Model**	2634,55	9	292,73	33,87	0,0006	significant
A-Temperature	190,25	1	190,25	22,02	0,0054	
B-Time	14,38	1	14,38	1,66	0,2535	
C-Activation ratio	143,43	1	143,43	16,60	0,0096	
AB	3,83	1	3,83	0,4429	0,5352	
AC	31,42	1	31,42	3,64	0,1149	
BC	52,73	1	52,73	6,10	0,0565	
A^2	86,02	1	86,02	9,95	0,0252	
B^2	104,43	1	104,43	12,08	0,0177	
C^2	1876,81	1	1876,81	217,17	< 0.0001	

Figure S1. ANOVA result for Response 1

ANOVA for Quadratic model

Response 2: Total pore volume
Transform: Square Root
Constant: 0

Source	Sum of Squares	df	Mean Square	F-value	p-value	
**Model**	1,98	9	0,2198	191,72	< 0.0001	significant
A-Temperature	0,1559	1	0,1559	135,97	< 0.0001	
B-Time	0,0214	1	0,0214	18,69	0,0075	
C-Activation ratio	0,0044	1	0,0044	3,85	0,1069	
AB	0,0014	1	0,0014	1,20	0,3225	
AC	0,0472	1	0,0472	41,18	0,0014	
BC	0,0382	1	0,0382	33,30	0,0022	
A^2	0,0734	1	0,0734	64,05	0,0005	
B^2	0,0343	1	0,0343	29,91	0,0028	
C^2	1,51	1	1,51	1317,36	< 0.0001	

Figure S2. ANOVA results for response 2

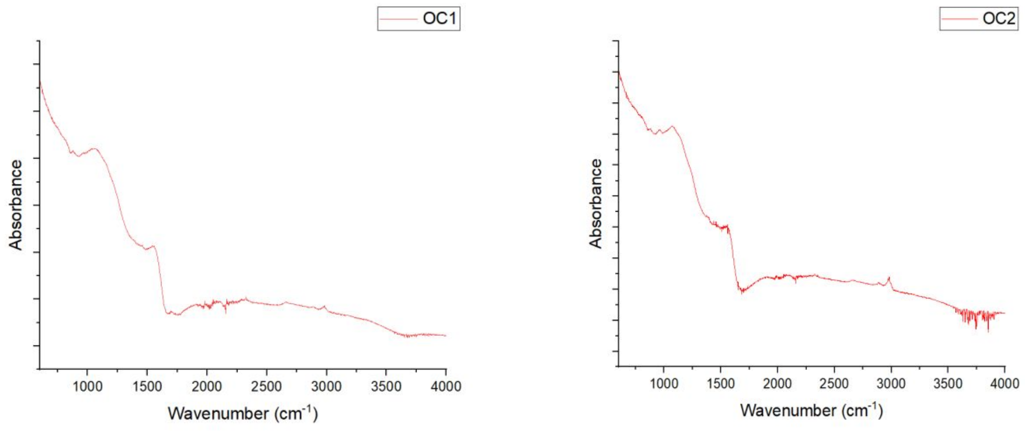

a) b)

Figure S3. FTIR spectra of a) OC1, b) OC2

References

(1) Chiu, Y. H.; Lin, L. Y. Effect of Activating Agents for Producing Activated Carbon Using a Facile One-Step Synthesis with Waste Coffee Grounds for Symmetric Supercapacitors. *J. Taiwan Inst. Chem. Eng.* **2019**, *101*. https://doi.org/10.1016/j.jtice.2019.04.050.

(2) Mohamedkhair, A. K.; Aziz, M. A.; Shah, S. S.; Shaikh, M. N.; Jamil, A. K.; Qasem, M. A. A.; Buliyaminu, I. A.; Yamani, Z. H. Effect of an Activating Agent on the Physicochemical Properties and Supercapacitor Performance of Naturally Nitrogen-Enriched Carbon Derived from Albizia Procera Leaves. *Arab. J. Chem.* **2020**, *13* (7). https://doi.org/10.1016/j.arabjc.2020.05.017.

(3) Martínez-Casillas, D. C.; Mascorro-Gutiérrez, I.; Arreola-Ramos, C. E.; Villafán-Vidales, H. I.;

Arancibia-Bulnes, C. A.; Ramos-Sánchez, V. H.; Cuentas-Gallegos, A. K. A Sustainable Approach to Produce Activated Carbons from Pecan Nutshell Waste for Environmentally Friendly Supercapacitors. *Carbon N. Y.* **2019**, *148*. https://doi.org/10.1016/j.carbon.2019.04.017.

(4) Xu, X.; Gao, J.; Tian, Q.; Zhai, X.; Liu, Y. Walnut Shell Derived Porous Carbon for a Symmetric All-Solid-State Supercapacitor. *Appl. Surf. Sci.* **2017**, *411*. https://doi.org/10.1016/j.apsusc.2017.03.124.

(5) Yu, K.; Zhu, H.; Qi, H.; Liang, C. High Surface Area Carbon Materials Derived from Corn Stalk Core as Electrode for Supercapacitor. *Diam. Relat. Mater.* **2018**, *88*. https://doi.org/10.1016/j.diamond.2018.06.018.

(6) Bai, X.; Quan, B.; Kang, C.; Zhang, X.; Zheng, Y.; Song, J.; Xia, T.; Wang, M. Activated Carbon from Tea Residue as Efficient Absorbents for Environmental Pollutant Removal from Wastewater. *Biomass Convers. Biorefinery* **2022**, No. 0123456789. https://doi.org/10.1007/s13399-022-02316-4.

(7) Chen, Z.; Zhang, M.; Wang, Y.; Yang, Z.; Hu, D.; Tang, Y.; Yan, K. Controllable Synthesis of Nitrogen-Doped Porous Carbon from Metal-Polluted Miscanthus Waste Boosting for Supercapacitors. *Green Energy Environ.* **2021**, *6* (6). https://doi.org/10.1016/j.gee.2020.07.015.

(8) Arampatzidou, A. C.; Deliyanni, E. A. Comparison of Activation Media and Pyrolysis Temperature for Activated Carbons Development by Pyrolysis of Potato Peels for Effective Adsorption of Endocrine Disruptor Bisphenol-A. *J. Colloid Interface Sci.* **2016**, *466*. https://doi.org/10.1016/j.jcis.2015.12.003.

(9) Apaydin-Varol, E.; Erülken, Y. A Study on the Porosity Development for Biomass Based Carbonaceous Materials. *J. Taiwan Inst. Chem. Eng.* **2015**, *54*. https://doi.org/10.1016/j.jtice.2015.03.003.

(10) Han, J.; Lee, J. H.; Roh, K. C. Herbaceous Biomass Waste-Derived Activated Carbons for Supercapacitors. *J. Electrochem. Sci. Technol.* **2019**, *9* (2). https://doi.org/10.33961/jecst.2018.9.2.157.

(11) Li, W.; Ding, Y.; Zhang, W.; Shu, Y.; Zhang, L.; Yang, F.; Shen, Y. Lignocellulosic Biomass for Ethanol Production and Preparation of Activated Carbon Applied for Supercapacitor. *J. Taiwan Inst. Chem. Eng.* **2016**, *64*. https://doi.org/10.1016/j.jtice.2016.04.010.

(12) Ahmed, M. B.; Hasan Johir, M. A.; Zhou, J. L.; Ngo, H. H.; Nghiem, L. D.; Richardson, C.; Moni, M. A.; Bryant, M. R. Activated Carbon Preparation from Biomass Feedstock: Clean Production and Carbon Dioxide Adsorption. *J. Clean. Prod.* **2019**, *225*. https://doi.org/10.1016/j.jclepro.2019.03.342.

(13) Mahamad, M. N.; Zaini, M. A. A.; Zakaria, Z. A. Preparation and Characterization of Activated Carbon from Pineapple Waste Biomass for Dye Removal. *Int. Biodeterior. Biodegrad.* **2015**, *102*. https://doi.org/10.1016/j.ibiod.2015.03.009.

(14) Kumar, A.; Jena, H. M. High Surface Area Microporous Activated Carbons Prepared from Fox Nut (Euryale Ferox) Shell by Zinc Chloride Activation. *Appl. Surf. Sci.* **2015**, *356*. https://doi.org/10.1016/j.apsusc.2015.08.074.

(15) Susanti, R. F.; Wiratmadja, R. G. R.; Kristianto, H.; Arie, A. A.; Nugroho, A. Synthesis of High Surface Area Activated Carbon Derived from Cocoa Pods Husk by Hydrothermal Carbonization and Chemical Activation Using Zinc Chloride as Activating Agent. *Mater. Today Proc.* **2022**. https://doi.org/10.1016/J.MATPR.2022.01.042.

(16) Wang, C.; Liu, T. Nori-Based N, O, S, Cl Co-Doped Carbon Materials by Chemical Activation of ZnCl2for Supercapacitor. *J. Alloys Compd.* **2017**, *696*. https://doi.org/10.1016/j.jallcom.2016.11.206.

(17) Wang, S.; Yu, J. Cotton-Derived Carbon Fibers with High Specific Capacitance by ZnCl2 Activation for Supercapacitor Application; 2016. https://doi.org/10.2991/icseee-16.2016.67.

(18) Du, X.; Zhao, W.; Ma, S.; Ma, M.; Qi, T.; Wang, Y.; Hua, C. Effect of ZnCl2 Impregnation Concentration on the Microstructure and Electrical Performance of Ramie-Based Activated Carbon Hollow Fiber. *Ionics (Kiel).* **2016**, *22* (4). https://doi.org/10.1007/s11581-015-1571-3.
